# Assessing the Association between Thermotolerant Coliforms in Drinking Water and Diarrhea: An Analysis of Individual–Level Data from Multiple Studies

**DOI:** 10.1289/EHP156

**Published:** 2016-05-10

**Authors:** James Hodge, Howard H. Chang, Sophie Boisson, Simon M. Collin, Rachel Peletz, Thomas Clasen

**Affiliations:** 1 Department of Environmental Health, and; 2 Department of Biostatistics and Bioinformatics, Rollins School of Public Health, Emory University, Atlanta, Georgia, USA; 3 Faculty of Infectious Diseases, London School of Hygiene and Tropical Medicine, London, United Kingdom; 4 School of Social and Community Medicine, University of Bristol, Bristol, United Kingdom; 5 Aquaya Institute, Nairobi, Kenya

## Abstract

**Background:**

Fecally contaminated drinking water is believed to be a major contributor to the global burden of diarrheal disease and a leading cause of mortality among young children. However, recent systematic reviews and results from blinded studies of water quality interventions have raised questions about the risk associated with fecally contaminated water, particularly as measured by thermotolerant coliform (TTC) bacteria, a WHO-approved indicator of drinking water quality.

**Objectives:**

We investigated the association between TTC in drinking water and diarrhea using data from seven previous studies.

**Methods:**

We obtained individual-level data from available field studies that measured TTC levels in household-drinking water and reported prevalence of diarrhea among household members during the days prior to the visit.

**Results:**

The combined data set included diarrhea prevalence for 26,518 individuals and 8,000 water samples from 4,017 households, yielding 45,052 observations. The odds of diarrhea increased for each log10 increase in TTC/100 mL by 18% (95% CI: 11, 26%) for children < 5 years old and 12% (95% CI: 8, 18%) for all ages. For all ages, the odds of diarrhea increased by 21%, 35% and 49% for those whose household water samples were from 11–100, 101–1,000, and > 1,000 TTC/100 mL, respectively compared to < 1 TTC/100 mL. We found no evidence of increased odds of diarrhea with contamination levels below 11 TTC/100 mL, either in adults or children.

**Conclusions:**

Our analysis of individual-level data shows increased risk of diarrhea with increasing levels of TTC in drinking water. These results suggest an association between fecally contaminated water and diarrheal disease and provides support for health-based targets for levels of TTC in drinking water and for interventions to improve drinking water quality to prevent diarrhea.

**Citation:**

Hodge J, Chang HH, Boisson S, Collin SM, Peletz R, Clasen T. 2016. Assessing the association between thermotolerant coliforms in drinking water and diarrhea: an analysis of individual level data from multiple studies. Environ Health Perspect 124:1560–1567; http://dx.doi.org/10.1289/EHP156

## Introduction

In 2013, diarrheal diseases caused an estimated 1.3 million deaths and were the fourth leading cause of years of life lost in developing countries (GBD 2013 Mortality and Causes of Death Collaborators 2015). For children under 5 years of age, diarrheal diseases were the fourth leading cause of death and caused approximately 800,000 deaths in 2010 (Liu et al. 2012). The majority of these deaths occurred in children under 5 years old in low-income countries, where diarrhea accounted for 12% of all child deaths in Africa and the Eastern Mediterranean and 11% of all child deaths in Southeast Asia (Liu et al. 2012).

Fecally contaminated drinking water quality, along with poor sanitation, hygiene, and water access, is generally believed to be major contributors to diarrheal disease (Fewtrell et al. 2005; Wolf et al. 2014). For this reason, WHO (World Health Organization) guidelines provide strict limits on the fecal contamination in drinking water supplies (WHO 2011). Along with *Escherichia coli*, thermotolerant coliforms (TTC) are a WHO-approved indicator of fecal contamination. Thermotolerant coliforms (sometimes referred to as fecal coliforms) are a class of bacteria comprising four species of coliforms that grow at elevated temperatures (44.5 ± 0.2°C). While consisting primarily of *E. coli*, TTC also includes *Klebisella, Enterobacter*, and *Citrobacter* species (Garcia-Armisen et al. 2007; Tallon et al. 2005). Like the *E. coli* indicator, WHO guidelines specify a limit of < 1 TTC/100 mL in public water supplies (WHO 2011).

Models using quantitative microbial risk assessment (QMRA) assume a dose–response relationship between fecal contamination and diarrhea (Enger et al. 2012; Howard et al. 2006). However, field studies have raised questions about the association between TTC and diarrheal disease. A recent systematic review of these studies by Gruber et al. (2014) found that while presence of *E. coli* was associated with an increased risk of diarrheal disease [RR: 1.54; 95% confidence interval (CI): 1.37, 1.74], presence of TTC was not (RR: 1.07; 95% CI: 0.79, 1.45). The review updated and expanded on a previous review by Gundry et al. (2004) that found an association between fecal indicator bacteria and cholera but not general diarrhea. Significantly, however, these reviews extracted and combined risk estimates from previous studies in a meta-analysis; they did not analyze individual level data.

We sought to explore the relation between TTC levels in drinking water and diarrheal disease by using individual-level data from multiple studies. To ensure comparability of data across studies and to minimize between-study heterogeneity, we only included studies that followed the same approaches to assessing diarrhea and sampling drinking water. This permitted an analysis of individual health conditions linked to a specific household drinking water sample.

## Methods

### Included Studies

The studies included in this analysis represent all the studies that we could identify that had available individual-level data and that used the same method for collection and analysis of water samples and the same method to ascertain cases of diarrheal disease (self-reported cases with a 7-day recall period) for children under 5 years and for householders of all ages. Table 1 and Table S1 provide details of each study. Accordingly, this is a convenience sample of studies and not the result of a comprehensive search strategy that would be undertaken in a systematic review executed in accordance with a prescribed protocol. Notably, none of the studies were designed or powered to investigate an association between water quality and diarrhea.

**Table 1:** Description of studies from which data were obtained and analyzed.

Reference	Country	Study design	Cited water quality measurement method	Case definition	Recall period for self-reported diarrhea
Clasen et al. 2005	Colombia	RCT	APHA	3+ Loose stools / 24 hr	7 Days
Clasen et al. 2006	Bolivia	RCT	APHA	3+ Loose stools / 24 hr	7 Days
Clasen et al. 2014	India	RCT	APHA	WHO^*b*^	7 Days
Boisson et al. 2009	Ethiopia	RCT	Not specified^*a*^	Local term “tekmat”	7 Days
Boisson et al. 2010	DR Congo	RCT	APHA	3+ Loose stools / 24 hr	7 Days
Peletz et al. 2011	Zambia	Cross-sectional	Not specified^*a*^	WHO^*b*^	7 Days
Peletz et al. 2012	Zambia	RCT	APHA	WHO^*b*^	7 Days
Abbreviations: APHA, American Public Health Association; RCT, randomized, controlled trial; WHO, World Health Organization. ^***a***^Though no specific method was cited, the method described in these articles followed the APHA membrane filtration method. ^***b***^The WHO definition of diarrhea is three or more loose or watery stools in a 24-hr period (WHO 2005).					

### Diarrhea Prevalence

In each study, diarrheal prevalence was obtained during the same household visit at which the water samples were collected. During the household visit, diarrhea prevalence over the preceding 7 days was ascertained by asking the female head of household or primary caretaker if any household members had diarrhea during the past 7 days. In each study diarrhea was defined according to the WHO definition as three or more loose or watery stools in a 24-hr period (WHO 2005), except the Ethiopia study that used a local definition.

### Water Quality

On the same visit to assess diarrheal prevalence, researchers obtained a drinking water sample by asking the female head of household what stored water householders were using for drinking at that time. Water samples were collected during household visits in either sterile 125 mL Nalgene bottles or sterile 125 mL WhirlPak bags containing a sodium thiosulfate tablet to neutralize any chlorine. All samples were stored on ice during transport and were processed within 4 hr to assess TTC levels. Microbiological assays were done using standard membrane filtration methods (APHA et al. 2005) with membrane lauryl sulphate medium. Samples were incubated at 44 ± 0.5°C for 18 hr. Following incubation, the number of colonies were counted and recorded as individual TTC and standardized to a count of TTC/100 mL of water.

### Data Extraction and Synthesis

Original data for each surveillance visit were obtained from the researchers of the previous studies. Diarrheal prevalence was obtained for individual householders; water quality data were obtained for the household and ascribed to each member of that household for that visit. The data were combined into one dataset retaining variables for age, household identifier, study identifier for each individual, and whether the individual reported having diarrhea over the preceding 7 days at each follow-up round. Each time point for each individual comprises a separate observation. Household level data on water quality [measured as colony forming units (CFU) of TTC per 100 mL of water] at each follow-up round were retained and matched to all individuals within a household. Because diarrhea generally varies over seasons (Das et al. 2014; Fisman 2007; Levy et al. 2009), an additional variable for the season (rainy/dry) was also included. Season was only recorded for two of the studies (Peletz et al. 2011, 2012). For the remainder, the season variable was assigned based on the date at which the observation occurred and data on rainfall from the National Climatic Data Center (https://www.ncdc.noaa.gov/cdo-web/) or Weatherbase (http://www.weatherbase.com/).

### Statistical Analysis

Analysis was done using multilevel logistic regression models with nested random intercepts to control for repeated measurements in individuals and clustering at the household level. The multilevel logistic regression model was estimated using the meqrlogit function in STATA. The estimation procedure is maximum likelihood-based following the algorithm by Pinheiro and Chao (2006). Water quality was included as the predictor variable as log_10_ transformed TTC/100 mL. The dependent variable was diarrheal disease as a binary outcome. The relationship between TTC and diarrheal disease was assessed separately for each study and again for the combined data set. Two models were fitted for each study: first for all ages and again for only children under 5 years of age. The first model was used to assess whether there was an apparent linear relationship between the number of TTC/100 mL and odds of diarrhea. It modeled log-odds of diarrheal disease using log_10_ TTC/100 mL as a continuous predictor to evaluate the odds of diarrhea for each log_10_, i.e. 10-fold, increase in TTC. A second model was fitted using WHO risk categories (WHO 1997) for five levels of contamination: < 1, 1–10, 11–100, 101–1,000, and > 1,000 TTC/100 mL. Adjusted odds ratios (ORs) were calculated for each category using < 1 TTC/100 mL as the reference group. Models that included all ages were controlled for age by including it as a categorical variable (< 5, 5–15, > 15) while the models limited to children under 5 years of age included age as a continuous variable. We conducted sub-group analysis by treatment status, but found that treatment status was not a significant predictor in any of the models, either for any of the studies individually or for all the studies combined (see Tables S2 and S3). Season was controlled for in all models except those for the Bolivia study (Clasen et al. 2006) which was conducted entirely within the dry season. Models fitted using the combined data set were also adjusted for study identifier. All data cleaning and management was done using SAS 9.4 and models were fitted using Stata 13. Graphics were generated using R (version 3.1.2; R Foundation for Statistical Computing).

### Sensitivity Analysis

A sensitivity analysis was also conducted to assess the effect of the Indian sanitation study (Clasen et al. 2014), which contributed the majority of the observations for this analysis. Each model was re-fitted using the combined data set but excluding data from the Indian sanitation study to determine the extent to which the overall outcome was influenced by that study.

### Ethics

The protocol for this study was approved by the Emory University Institutional Review Board (IRB00079426). Each of the studies from which data were obtained was approved by the Ethics Committee of the London School of Hygiene and Tropical Medicine and by local ethics committees in the countries in which they were conducted.

## Results

### Included Studies

Individual level data were drawn from seven studies of water quality and sanitation conditions and diarrheal disease (Table 1). Five of the studies were randomized controlled trials of household water treatment interventions (Boisson et al. 2009, 2010; Clasen et al. 2005, 2006; Peletz et al. 2012). One study was a randomized controlled trial of a sanitation intervention (Clasen et al. 2014). One study followed a cross-sectional design (Peletz et al. 2011). One of the randomized controlled trials (Boisson et al. 2009) did not include usable water quality data for the follow-up time period, so only baseline measurements were used, and it was treated as a cross-sectional study. All were conducted among rural, low-income populations, except for the Zambia studies, which were in a peri-urban setting where the study population was limited to households with children < 2 years whose mothers were HIV positive. For the randomized controlled trials, data from both the intervention and control groups was used. Additional details concerning the included studies and their respective methods are included in Table S1.

### Population and Demographics

Table 2 shows the distribution of observations among the studies, the age distributions, and observations per season for each study, individually and combined. The combined data set included data for 26,518 individuals from 4,017 households. The Indian sanitation trial contributed the majority of the individuals (79.3%) and households (72.2%). Overall, 20.9% of the study population consisted of children under 5 years. Distribution among the age categories was variable between studies as well with the Zambia RCT having the highest proportion of children under 5 (34%) and the Ethiopia study having the lowest (12.9%). The combined data set included 7-day diarrhea prevalence on 26,518 individuals and 8,000 water samples from 4,017 households. This yielded 45,052 observations of diarrhea prevalence linked with a household drinking water sample.

**Table 2:** Characteristics of individual studies included in aggregated data set.

Characteristics	Cross-sectional		Randomized controlled trials					Combined	
	Zambia CS	Ethiopia	Colombia	Bolivia	Zambia RCT	DR Congo	India	All studies	All exc. India
Population
Total households	254	314	137	59	120	231	2,902	4,017	1,115
Total individuals	1,246	1,516	681	317	615	1,104	21,039	26,518	5,479
Age
Mean ± SD	15.6 ± 14.3	21.8 ± 18.3	19.0 ± 16.8	20.8 ± 19.1	14.8 ± 14.3	21.6 ± 18.5	26.8 ± 20.9	26.5 ± 20.4	19.3 ± 17.2
Median	11	16	14	14	10	16	26	25	14
< 5 (%)	374 (31.9)	196 (12.9)	142 (20.9)	60 (19.0)	193 (34.0)	185 (16.8)	4,298 (20.8)	5,448 (20.9)	1,150 (21.5)
5–15 (%)	307 (26.1)	534 (35.2)	231 (34.0)	108 (34.2)	155 (27.3)	348 (31.6)	2,723 (13.1)	4,406 (16.9)	1,683 (31.4)
> 16 (%)	493 (42.0)	786 (51.8)	307 (45.1)	148 (46.8)	220 (38.7)	568 (51.6)	13,691 (66.1)	16,213 (62.2)	2,522 (47.1)
Follow-up rounds	—	—	3	2	12	14	10	14	14
Diarrhea
Baseline DD prevalence	13.2	8.52	21.8	—	11.9	11.8	—	12.5	12.5
Mean DD prevalence ± SD	—	—	7.3 ± 3.2	3.9 ± 0.1	2.3 ± 1.5	2.8 ± 1.5	4.4 ± 2.4	3.7 ± 2.3	2.9 ± 1.8
Total DD observations	1,246	1,517	2,736	634	7,588	3,970	179,690	197,381	17,691
Observations with DD and WQ	1,083	1,159	1,977	542	6,131	3,681	30,479	45,052	14,573
Season
Dry (%)	174 (14.0)	1,470 (96.9)	597 (21.8)	634 (100)	3,430 (50.4)	1,970 (49.6)	25,321 (14.1)	33,596 (17.1)	8,275 (48.9)
Rainy (%)	1,072 (86.0)	47 (3.1)	2,139 (78.2)	0 (0.0)	3,375 (49.6)	2,000 (50.4)	154,369 (85.9)	163,002 (82.9)	8,633 (51.1)
Study arm
Intervention	—	748 (49.3)	1,676 (61.3)	408 (67.0)	3,765 (49.6)	1,928 (48.6)	88,581 (49.4)	97,106 (49.3)	7,377 (48.7)
Control	—	769 (50.7)	1,060 (38.7)	201 (33.0)	3,823 (50.4)	2,042 (51.4)	90,785 (50.6)	99,935 (50.7)	7,785 (51.3)
Abbreviations: CS, cross-sectional; DD, diarrheal disease; RCT, randomized, controlled trial; WQ, water quality.									

### Diarrhea Prevalence

Table 3 shows the distribution of diarrhea for each study by age category, TTC category, and season, and the overall prevalence of diarrhea in each category. In all studies individually and in the combined data, children under 5 had the highest prevalence of diarrhea over the length of the studies. Prevalence among categories of TTC were variable between studies but in general, prevalence increased with increasing TTC counts. In the combined data, prevalence increased from 3.9% for < 1 TTC/100 mL to 5.2% for > 1,000 TTC/100 mL. Prevalence of diarrhea cases by season was variable across studies and in part reflected the distribution of observations among seasons. However, when combined, there was strong evidence (*p* < 0.01) of a difference in prevalence of cases in the rainy (4.6%) and dry seasons (4.0%) though the overall difference was small.

**Table 3:** Total cases of diarrhea, total observations, and prevalence for each study by category.

Category	Cases/observations (prevalence)							
	Colombia	Bolivia	Zambia CS	Zambia RCT	Ethiopia	DR Congo	India	All studies
Total	281/2,441 (11.5%)	23/592 (3.9%)	163/1,236 (13.2%)	222/6,671 (3.3%)	128/1,500 (8.5%)	187/3,690 (5.1%)	6,800/156,357 (4.3%)	7,804/164,683 (4.5%)
Age category
< 5	130/501 (25.9%)	16/114 (14.0%)	83/372 (22.3%)	167/2,045 (8.2%)	28/194 (14.4%)	87/624 (13.9%)	2,616/26,446 (9.9%)	3,127/30,296 (10.3%)
5–15	85/822 (10.3%)	4/205 (2.0%)	25/306 (8.2%)	19/1,721 (1.1%)	35/533 (6.6%)	29/1,214 (2.4%)	901/26,733 (3.4%)	1,098/31,534 (3.5%)
> 15	64/1,105 (5.8%)	3/271 (1.1%)	48/488 (9.8%)	32/2,396 (1.3%)	65/773 (8.4%)	71/1,843 (3.9%)	3,283/103,171 (3.2%)	3,566/110,047 (3.2%)
TTC category
< 1	41/590 (6.9%)	11/345 (3.2%)	44/341 (12.9%)	41/2,111 (1.9%)	11/219 (5.0%)	37/1,053 (3.5%)	384/9,749 (3.9%)	569/14,408 (3.9%)
1–10	24/342 (7.0%)	1/50 (2.0%)	8/97 (8.2%)	23/986 (2.3%)	9/69 (13.0%)	14/430 (3.3%)	70/1,901 (3.7%)	149/3,875 (3.8%)
11–100	74/602 (12.3%)	5/97 (5.2%)	14/148 (9.5%)	39/1,232 (3.2%)	24/370 (6.5%)	20/265 (7.5%)	211/5,466 (3.9%)	387/8,180 (4.7%)
101–1,000	54/452 (11.9%)	4/51 (7.8%)	55/401 (13.7%)	68/1,314 (5.2%)	39/383 (10.2%)	76/1,394 (5.5%)	299/7,159 (4.2%)	595/11,154 (5.3%)
> 1,000	—	—	27/160 (16.9%)	50/994 (5.0%)	15/118 (12.7%)	40/548 (7.3%)	286/6,206 (4.6%)	418/8,026 (5.2%)
*p*-Value^*a*^	0.002	0.321	0.206	< 0.001	0.023	0.001	0.167	< 0.001
Season
Dry	74/398 (18.6%)	23/592 (3.9%)	16/174 (9.2%)	128/3,378 (3.8%)	128/1,453 (8.8%)	62/1,818 (3.4%)	776/22,023 (3.5%)	1,207/29,836 (4.0%)
Rainy	207/2,043 (10.1%)	—	147/1,062 (13.8%)	94/3,293 (2.9%)	0/47 (0.0%)	125/1,872 (6.7%)	6,024/134,334 (4.5%)	6,597/142,651 (4.6%)
*p*-Value^*a*^	< 0.001	—	0.093	0.033	0.033	< 0.001	< 0.001	< 0.001
Study arm
Intervention	157/1,525 (10.3%)	12/392 (2.7%)	—	94/3,467 (2.7%)	67/741 (9.0%)	87/1,782 (4.9%)	3,332/77,033 (4.3%)	3,749/84,940 (4.4%)
Control	124/916 (13.5%)	11/195 (5.6%)	—	128/3,204 (4.0%)	61/759 (8.0%)	100/1,908 (5.2%)	3,468/79,324 (4.4%)	4,055/87,542 (4.6%)
*p*-Value^*a*^	0.019	0.13	—	0.003	0.486	0.619	0.652	0.029
Abbreviations: CS, cross-sectional; RCT, randomized controlled trial. ^***a***^*p*-Values for chi-square test of homogeneity.								

### Water Quality

Table 4 shows water quality data for each study separately and for the combined data. Water quality was highly variable across and within studies. The DR Congo study had the highest arithmetic mean with 1,548 TTC/100 mL. Bolivia had the lowest arithmetic mean with 35 TTC/100 mL. All of the studies had highly skewed TTC data as seen by the difference between the mean and median values. Thus, values of TTC were log_10_ transformed prior to analysis. The majority of studies had high numbers of households with < 1 TTC/100 mL. This result is not unexpected considering five of the seven studies were assessing water treatment technologies and the data includes households in both the intervention and control arms of the studies. However, all the studies also had at least 10% of the observations in the highest category. For the combined data set, 30.9% of samples had < 1 TTC/100 mL and 17.2% were > 1,000 TTC/100 mL.

**Table 4:** Water quality measurements by study.

Reference	Country	*N*	CFU TTC/100 mL		Log_10_ TTC		Number of households per TTC category (%)				
			Mean ± SD	Median	Mean ± SD	Median	< 1	1–10	11–100	101–1,000	> 1,000
Clasen et al. 2005	Colombia	401	77.8 ± 115.8	17	1.2 ± 0.9	1.2	104 (25.9)	71 (17.7)	127 (31.7)	99 (24.7)	—
Clasen et al. 2006	Bolivia	101	35.3 ± 87.7	0	0.6 ± 0.9	0.0	64 (63.4)	9 (8.9)	16 (15.8)	12 (11.9)	—
Peletz et al. 2011	Zambia CS	234	700.3 ± 2130.4	74	1.6 ± 1.3	1.9	71 (30.3)	21 (9.0)	29 (12.4)	84 (35.9)	29 (12.4)
Peletz et al. 2012	Zambia RCT	1,313	668.7 ± 1802.9	20	1.4 ± 1.3	1.3	421 (32.1)	196 (14.9)	240 (18.3)	261 (19.9)	195 (14.9)
Boisson et al. 2009	Ethiopia	234	451.4 ± 1792.1	85	1.7 ± 1.1	1.9	47 (20.1)	12 (5.1)	72 (30.8)	79 (33.8)	24 (10.3)
Boisson et al. 2010	DR Congo	815	1548.5 ± 5721.8	140	1.6 ± 1.4	2.2	236 (29.0)	99 (12.1)	56 (6.9)	319 (39.1)	105 (12.9)
Clasen et al. 2014	India	4,902	686.8 ± 1147.6	60	1.7 ± 1.3	1.8	1,528 (31.2)	300 (6.1)	874 (17.8)	1,180 (24.1)	1,020 (20.8)
Overall		8,000	726.4 ± 2235.6	47	1.6 ± 1.3	1.7	2,471 (30.9)	708 (8.8)	1,414 (17.7)	2,034 (25.4)	1,373 (17.2)
Abbreviations: CFU, colony forming unit; CS, cross-sectional; RCT, randomized, controlled trial; TTC, thermotolerant coliform.											

### TTC-Diarrhea Association

**Figure 1:**
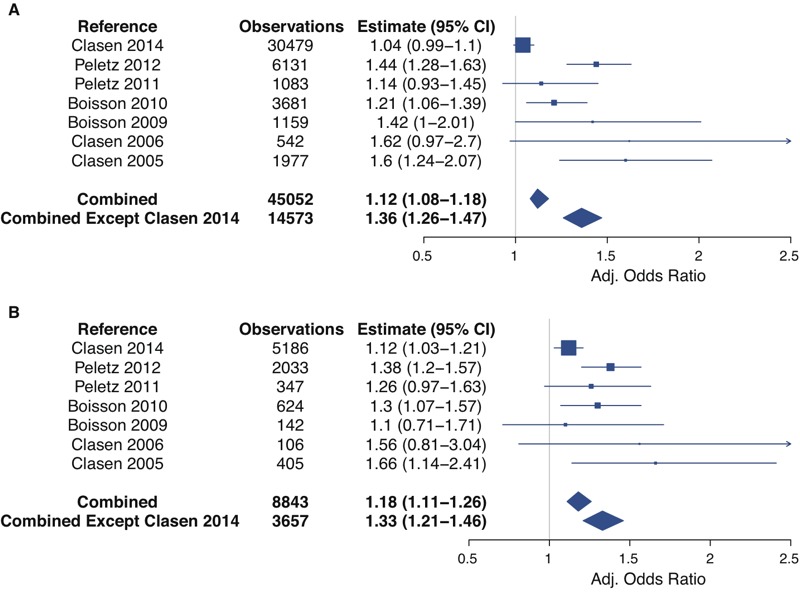
Forest plot of adjusted odds ratios from multi-level logistic regression model with log_10_ TTC as a continuous predictor for all ages (*A*) and for children under 5 (*B*). All ages models are adjusted for age as a categorical variable (< 5, 5–15, > 15) and season. Models for children under 5 are adjusted for age as a continuous variable and season. The summary measures are also adjusted for study location. Study-specific point estimates are proportional to the number of observations.

The combined dataset showed that an increase in TTC results in increased odds of diarrhea for all ages and for children under 5. Table 5 and Figure 1 show adjusted ORs for a 1-log increase in TTC/100 mL of household drinking water. Of the seven studies, three (Colombia, Zambia RCT, and DR Congo) showed convincing evidence (*p* ≤ 0.01) that increasing the level of TTC in drinking water was associated with increased odds of diarrhea (point estimates indicating 20–60% higher odds with 95% CI from 1.06 to 2.07); four other studies (Ethiopia, Bolivia, Zambia CS, and India) showed weaker evidence of this association. A similar pattern was seen for children under 5 years old, with four studies (Colombia, Zambia RCT, DR Congo, and India) showing good evidence of an association (point estimates indicating 12–66% higher odds with 95% CI from 1.03 to 2.41), and one (Zambia CS) showing weaker evidence. When combined, there is a 12% (95% CI: 8%, 18%) greater odds of diarrhea for each 1-log increase in TTC/100 mL for all ages. For children under 5 the effect is larger with an 18% (95% CI: 11%, 26%) increase in odds of diarrhea for each log_10_ increase in TTC/100 mL.

**Table 5:** Adjusted odds ratios of diarrhea for log_10_ TTC/100 mL.

Reference	Country	All ages		Children < 5	
		Adj. OR (95% CI)	*p*-Value	Adj. OR (95% CI)	*p*-Value
Peletz et al. 2011	Zambia CS	1.14 (0.93, 1.42)	0.192	1.26 (0.97, 1.63)	0.087
Boisson et al. 2009	Ethiopia	1.42 (1.00, 2.01)	0.049	1.10 (0.71, 1.71)	0.657
Clasen et al. 2006	Bolivia	1.62 (0.97, 2.70)	0.063	1.56 (0.81, 3.04)	0.186
Clasen et al. 2005	Colombia	1.60 (1.24, 2.07)	< 0.001	1.66 (1.14, 2.41)	0.008
Peletz et al. 2012	Zambia RCT	1.44 (1.28, 1.63)	< 0.001	1.38 (1.20, 1.57)	< 0.001
Boisson et al. 2010	DR Congo	1.21 (1.06, 1.39)	0.006	1.30 (1.07, 1.57)	0.007
Clasen et al. 2014	India	1.04 (0.99, 1.10)	0.099	1.12 (1.03, 1.21)	0.010
Combined		1.12 (1.08, 1.18)	< 0.001	1.18 (1.11, 1.26)	< 0.001
Combined except India		1.36 (1.26, 1.47)	< 0.001	1.33 (1.21, 1.46)	< 0.001
Abbreviations: CS, cross-sectional; RCT, randomized, controlled trial. Note: All studies were adjusted for categorical ages (< 5, 5–15, > 15) and season (rainy/dry) except Bolivia, which was adjusted only for age because all observations occurred in the dry season.					

### Categorical Analysis

**Figure 2:**
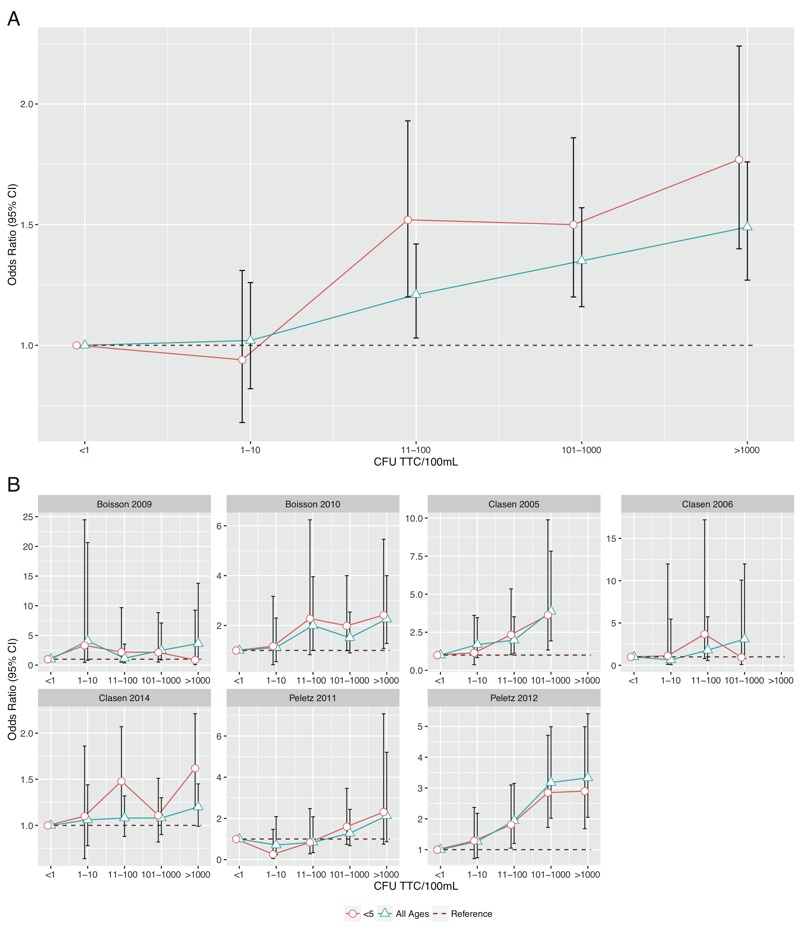
Adjusted odds ratios and 95% confidence intervals for WHO risk categories (with < 1 TTC/100 mL as references) for (*A*) all studies combined and (*B*) each study individually.

Table 6 shows the adjusted ORs for the increasing categories of TTC/100 mL with the lowest category (< 1 TTC/100 mL) as the reference group. In each study individually and in the combined analysis, there is a positive relationship between the higher exposure categories and odds of diarrhea in the past 7 days for all ages and for children under 5 years. However, neither the individual studies nor the combined data show evidence of increased odds of diarrhea below 11 TTC/100 mL. For the combined data, the OR of diarrhea in the preceding 7 days for the 1–10 TTC category compared to the < 1 category is 1.02 (95% CI: 0.82, 1.26) for all ages and 0.94 (95% CI: 0.68, 1.31) for children under 5. However, for the 11–100 TTC category and for all higher categories, for all ages and for children under 5, the ORs show evidence of a positive association (Figure 2).

**Table 6:** Diarrhea odds ratios (95% CI) for categories of thermotolerant coliforms in household drinking water.

Reference	Country	TTC/100 mL drinking water				
		< 1^*a*^	1 to 10	11 to 100	101 to 1,000	> 1,000
Peletz et al. 2011	Zambia CS
All ages		—	0.71 (0.24, 2.09)	0.84 (0.34, 2.08)	1.28 (0.68, 2.44)	2.13 (0.87, 5.21)
Children < 5		—	0.26 (0.05, 1.47)	0.84 (0.28, 2.48)	1.60 (0.74, 3.46)	2.31 (0.75, 7.08)
Boisson et al. 2009	Ethiopia
All ages		—	4.09 (0.81, 20.65)	1.16 (0.38, 3.54)	2.49 (0.87, 7.10)	3.64 (0.96, 13.79)
Children < 5		—	3.33 (0.45, 24.48)	2.20 (0.49, 9.68)	2.15 (0.52, 8.85)	0.87 (0.08, 9.26)
Clasen et al. 2006	Bolivia
All ages		—	0.64 (0.08, 5.46)	1.79 (0.56, 5.74)	3.10 (0.80, 11.99)	—
Children < 5		—	1.13 (0.11, 11.97)	3.69 (0.79, 17.19)	0.95 (0.09, 10.06)	—
Clasen et al. 2005	Colombia
All ages		—	1.69 (0.83, 3.46)	1.98 (1.12, 3.52)*	3.88 (1.93, 7.83)*	—
Children < 5		—	1.15 (0.37, 3.61)	2.34 (1.03, 5.35)*	3.62 (1.32, 9.89)*	—
Peletz et al. 2012	Zambia RCT
All ages		—	1.27 (0.74, 2.18)	1.94 (1.20, 3.15)*	3.18 (2.02, 4.99)*	3.33 (2.05, 5.41)*
Children < 5		—	1.29 (0.71, 2.37)	1.81 (1.05, 3.10)*	2.85 (1.72, 4.71)*	2.90 (1.68, 4.99)*
Boisson et al. 2010	DR Congo
All ages		—	1.12 (0.55, 2.30)	2.00 (1.01, 3.96)*	1.51 (0.90, 2.54)	2.26 (1.28, 4.00)*
Children < 5		—	1.17 (0.43, 3.17)	2.28 (0.83, 6.24)	1.99 (0.99, 4.00)	2.42 (1.08, 5.46)*
Clasen et al. 2014	India
All ages		—	1.06 (0.78, 1.44)	1.08 (0.88, 1.32)	1.08 (0.90, 1.30)	1.20 (0.99, 1.45)
Children < 5		—	1.10 (0.65, 1.86)	1.48 (1.06, 2.07)*	1.11 (0.82, 1.51)	1.62 (1.19, 2.21)*
Combined
All ages		—	1.02 (0.82, 1.26)	1.21 (1.03, 1.42)*	1.35 (1.16, 1.57)*	1.49 (1.27, 1.76)*
Children < 5		—	0.94 (0.68, 1.31)	1.52 (1.20, 1.93)*	1.50 (1.20, 1.86)*	1.77 (1.40, 2.24)*
Combined except Clasen et al. 2014
All ages		—	1.21 (0.88, 1.68)	1.66 (1.25, 2.19)*	2.30 (1.76, 2.99)*	2.97 (2.17, 4.08)*
Children < 5		—	1.02 (0.67, 1.56)	1.81 (1.29, 2.55)*	2.28 (1.66, 3.13)*	2.38 (1.62, 3.51)*
Abbreviations; CS, cross-sectional; RCT, randomized, controlled trial; TTC, thermotolerant coliforms. ^***a***^< 1 TTC/100 mL was used as the reference group for calculating ORs. *Significant at α = 0.05.						

### Sensitivity Analysis

The Indian sanitation trial contributed the majority of the observations to the combined data set and it had a clear effect on the overall outcome. By itself, the adjusted OR for all ages for the Indian sanitation study was positive but the 95% CI included the null (1.04, 95% CI: 0.99, 1.10, *p* = 0.1). Excluding the Indian sanitation study from the overall analysis increases the adjusted OR for all ages for the combined data by 3-fold from 1.12 (95% CI: 1.08, 1.18) to 1.36 (95% CI: 1.26, 1.47) (Table 5).

## Discussion

Our analysis of individual-level data from seven comparable studies showed a significant increase in odds of diarrhea with increasing log_10_ TTC in drinking water. The observed effect was stronger for children under 5, the population group most vulnerable to diarrheal disease. The increasing odds observed in the categorical analysis as contamination increased suggests a dose-response effect that is consistent with the WHO risk categories (WHO 1997). At the same time, we found no evidence of increased odds of diarrhea with contamination levels between 1 and 10 TTC/100 mL, the category designated as “low-risk.”

Our findings of an association between TTC levels in drinking water and recent diarrheal disease are in contrast to the conclusions from previous systematic reviews. One possible reason is the different methodology used. Though we also drew data from multiple previous studies, we combined and analyzed individual-level data to estimate the effect of TTC on diarrhea at the individual level rather than analyzing group effect estimates to reach a single summary effect. This method provides several advantages over analysis of previously published summary estimates, and can result in less biased summary estimates (Riley et al. 2010; Thomas et al. 2014). In this case, it allowed for standardization of data and statistical analysis across studies, consistent adjustment for confounders and a larger sample size that allowed for analysis of different age groups. However, this method is limited in that it only includes studies where individual-level data were obtained rather than including all studies that reported a summary estimate. A second possible explanation is the range of methods used to measure diarrhea risk in the studies included in the previous reviews, the variety in study design, and the quality of the analysis conducted in those studies (Stewart and Parmar 1993). The risk measures in the studies included in the most recent review (Gruber et al. 2014; Gundry et al. 2004) included prevalence ratios, incidence density ratios, and odds ratios. The reported estimates also used a variety of levels of TTC as comparisons for the reported estimates and typically compared only two groups. Additionally, the included studies were either case-control or cohort studies and none were randomized, controlled trials. Finally, five of the seven studies did not control for covariates and it is possible that confounding factors may have influenced the reported estimates.

This study is subject to the same limitations as many studies of water quality and diarrheal disease. First, except for the DR Congo study, which was blinded, all of the studies included in this analysis followed open study designs and relied on subjective outcomes (self-reported diarrhea). Because the households knew whether they were part of the intervention group, their reporting of diarrhea prevalence may have been biased. Diarrhea prevalence could have suffered from reporting bias, however, study participants were blinded to TTC counts in their drinking water, an objective measure. This minimizes the risk of bias in assessing the link between diarrhea and TTC. The included studies also consistently used a 7-day recall period that has been recommended by some researchers to minimize recall bias (Arnold et al. 2013). Second, the different studies used different values when assigning quantities when the CFU were too numerous to count. However, the values assigned are typically lower than the actual likely value essentially placing a cap on the category that the water quality measurement can fall within. This cap means that there is potential exposure misclassification with high contamination (> 1,000 TTC/100 mL) classified as moderate contamination (101–1,000 TTC/100 mL).

More seriously, there is a time lag inherent in measuring water quality and 7-day recall of diarrhea: there can be no assurances that the drinking water measured will be the same as that present in the home on the days of the diarrhea episode. On the one hand, householders with diarrhea may be shedding fecal indicator bacteria that could yield higher levels of TTC in stored drinking water than that consumed at the onset of disease. On the other hand, householders with diarrhea may be more likely to procure their water from higher quality sources or to boil or otherwise treat their water, so that the subsequently collected sample would be of higher quality than consumed at the time of disease onset. These uncertainties are inherent in the study design. However, a recent study by Luby et al. (2015) specifically addressed this issue and found that *E. coli* contamination in drinking water was associated with increased prevalence of subsequent diarrhea (prevalence ratio: 1.14, 95% CI: 1.05, 1.23), providing further support for the increased risk from drinking contaminated water. Finally, we were unable to adjust for covariates that may have influenced diarrhea status such as sanitation and hygiene, socioeconomic status, and education and these external factors may bias the results.

Despite these limitations, the results of this study provide strong evidence of an association between the level of TTC in drinking water and the odds of diarrhea over the previous 7 days. This has important implications for research and policy. While differences in results between blinded and open trial designs have raised doubts about the reliability of subjective outcomes in non-blinded studies, our results are consistent with systematic reviews assessing the health impact of improving water quality (Clasen et al. 2007; Fewtrell et al. 2005; Wolf et al. 2014) and models using QMRA (Brown and Clasen 2012; Enger et al. 2012; Howard et al. 2006; Machdar et al. 2013). Although studies increasingly endeavor to use objective measures of disease outcomes, the options for assessing diarrhea are still limited and our results suggest that self-reports should not necessarily be dismissed. Our results also provide data that can be used to develop QMRA models to assess the relationship between fecal contamination and diarrheal disease.

Our results have even more important policy implications. First, the evidence of increasing odds of diarrhea with increasing levels of TTC challenge the conclusions of the GBD study (Lim et al. 2012) and Engell and Lim (2013) review that water quality is not a risk factor for diarrheal disease. On the other hand, they are consistent with more recent reviews that conclude that water quality interventions are protective against diarrhea, particularly when they improve water quality through the point of use (Clasen et al. 2015; Wolf et al. 2014). Second, our results provide support for the continued use of TTC as an indicator of health risks associated with fecal contamination of drinking water. At the same time, they suggest the need for further research on whether contamination levels up to 10 TTC/100 mL actually present no risk of diarrhea. Previous Sphere Project standards set the minimum at 10 TTC/100 mL before being reduced to no detectable fecal coliforms per 100 mL in 2004 (Sphere Project 2000, 2004).

## Conclusions

While fecal contamination of drinking water is generally believed to represent a major health risk, pooled estimates of effect from systematic reviews have raised questions about the relationship between diarrhea and TTC, a widely-used fecal indicator bacteria. The lack of a protective effect on diarrhea from blinded trials of water quality interventions has also raised questions about the role of water quality as a risk factor. Our analysis of comparable data from more than 45,000 observations linking recent diarrhea prevalence with household water samples provides evidence of a dose-response relationship between diarrhea and fecal contamination household drinking water as measured by TTC. These results support both the continued use of health-based targets for levels of TTC in drinking water and interventions to improve drinking water quality to prevent diarrhea. This study has limited potential, however, for causal inference, and further research is necessary to characterize the relationship between fecal contamination of drinking water as measured by TTC and diarrheal disease.

## Supplemental Material

(134 KB) PDF
