# Supplementary material for: Assessing the Association between Thermotolerant Coliforms in Drinking Water and Diarrhea: An Analysis of Individual–Level Data from Multiple Studies
Source: Environ Health Perspect. 2016 May 10;124(10):1560–7. doi: 10.1289/EHP156 (PMC5047765; doi:10.1289/EHP156)
Supplement: (134 KB) PDF [file EHP156.s001.acco.pdf]

Environ Health Perspect
DOI: 10.1289/EHP156

Note to readers with disabilities: *EHP* strives to ensure that all journal content is accessible to all readers. However, some figures and Supplemental Material published in *EHP* articles may not conform to 508 standards due to the complexity of the information being presented. If you need assistance accessing journal content, please contact ehp508@niehs.nih.gov. Our staff will work with you to assess and meet your accessibility needs within 3 working days.

Supplemental Material

Assessing the Association between Thermotolerant Coliforms in Drinking Water and Diarrhea: An Analysis of Individual Level Data from Multiple Studies

James Hodge, Howard H. Chang, Sophie Boisson, Simon M. Collin, Rachel Peletz, and Thomas Clasen

Table of Contents

Table S1: Details of Methods for Studies from which Data was Obtained

Table S2: Adjusted Odds Ratios of Diarrhea for Log 10 TTC / 100ml by Treatment Status

Table S3: Adjusted Odds Ratios for Diarrhea for Log 10 TTC / 100mL

Environ Health Perspect
DOI: 10.1289/EHP156

Table S1: Details of Methods for Studies from which Data was Obtained

Author	Study	Study Design	Follow-up Duration	Frequency of Follow-up	Study Time-Frame	Intervention	Additional Details
Peletz, et al.	Zambia CS	Cross-sectional	-	-	Nov. 2009 - Apr. 2010	-	Water samples were collected and diarrhea cases for the preceding seven days were obtained during each household visit.
Boisson, et al.	Ethiopia	RCT	5 months	Fortnightly	Oct. 2007 - June 2008	Portable membrane filter	Households were visited 10 times in total and diarrhea cases for the preceding 7 days were recorded. Monthly, a 25% random subsample of filters was selectetd and water samples were obtained.
Clasen, et al.	Bolivia	RCT	5 months	Monthly	June - Oct. 2003	Ceramic filter	Each participating household was visited 5 times during follow up. Water samples were collected during the first two household visits. At each visit, the female head of household was about diarrhea status for all household members for the 7 days preceding the visit.
Clasen, et al.	Colombia	RCT	6 months	Six week intervals		Ceramic filter	Each participating household was visited 4 times at approximately 6 week intervals. Water samples and diarrhea prevalence over the previous seven days were recorded at each visit.
Boisson, et al.	DR Congo	RCT	12 months	Monthly	Apr. 2008 - July 2009	Membrane filter	Female heads of household were interviewed at each follow-up visit and any diarrhea cases over the past 7 days were recorded. At each follow-up point, water samples were collected from 60 households (30 in each arm).
Peletz, et al.	Zambia RCT	RCT	7-12 months	Monthly	Apr. 2010 - July 2011	Membrane filter	Households were visited 7 to 12 times depending on when they were enrolled. Water samples were collected and diarrhea cases over the preceding 7 days were recorded at each household visit.
Clasen, et al.	India	RCT	28 months	Every 3 months	June 2011 - Oct. 2013	Latrine promotion and construction	Diarrhea cases for the previous 7 days were recorded at each household visit. At each follow-up period, 20% of participating households were randomly selected for sampling and testing of household drinking water.

Table S2: Adjusted Odds Ratios of Diarrhea for Log 10 TTC / 100ml by Treatment Status[a]

		All Ages		Children Under 5	
Study		Adj. OR (95% CI)	p-value	Adj. OR (95% CI)	p-value
Ethiopia					
	Intervention	1.26 (0.65, 2.46)	0.488	1.32 (0.57, 3.11)	0.514
	Control	1.47 (0.99, 2.19)	0.059	0.99 (0.54, 1.80)	0.969
Bolivia[b]					
	Intervention				
	Control	2.90 (0.47, 17.89)	0.252	0.82 (0.09, 7.91)	0.865
Colombia					
	Intervention	1.71 (1.24, 2.36)	0.001	1.93 (1.21, 3.07)	0.006
	Control	1.55 (0.87, 2.78)	0.137	0.88 (0.38, 2.09)	0.786
Zambia RCT					
	Intervention	1.89 (1.56, 2.28)	<0.001	1.68 (1.34, 2.11)	<0.001
	Control	1.12 (0.92, 1.36)	0.261	1.08 (0.87, 1.34)	0.483
DR Congo					
	Intervention	1.37 (1.14, 1.64)	0.001	1.60 (1.21, 2.11)	0.001
	Control	1.15 (0.87, 1.51)	0.319	1.20 (0.84, 1.70)	0.318
India					
	Intervention	1.04 (0.97, 1.12)	0.276	1.12 (0.99, 1.27)	0.072
	Control	1.05 (0.97, 1.12)	0.219	1.11 (0.99, 1.25)	0.064
Combined[c]					
	Intervention	1.17 (1.10, 1.25)	<0.001	1.26 (1.15, 1.39)	<0.001
	Control	1.07 (1.01, 1.14)	0.021	1.09 (1.00, 1.19)	0.041
Combined Except India					
	Intervention	1.64 (1.46, 1.84)	<0.001	1.63 (1.41, 1.91)	<0.001
	Control	1.14 (1.02, 1.29)	0.021	1.09 (0.95, 1.25)	0.221

RCT = randomized, controlled trial

[a]All models were adjusted for categorical ages (<5, 5-15, >15) and season (rainy/dry) except Bolivia which was adjusted only for age because all observations occurred in the dry season.

[b]Models for the Intervention group for the Bolivia study did not have sufficient numbers in each group to produce a reliable estimate.

[c]Combined data includes the Zambia Cross-Sectional Study data as part of the control group.

Table S3: Adjusted Odds Ratios for Diarrhea for Log 10 TTC / 100mL[a]

	All Ages			Children <5		
Study	Adj. OR (95% CI)	p-value	Treatment Status p-value[b]	Adj. OR (95% CI)	p-value	Treatment Status p-value[b]
Ethiopia	1.42 (1.00, 2.01)	0.049	0.767	1.10 (0.71, 1.71)	0.670	0.491
Bolivia	2.02 (0.48, 8.41)	0.334	0.736	0.78 (0.14, 4.24)	0.773	0.387
Colombia	1.69 (1.28, 2.24)	<0.001	0.497	1.59 (1.04, 2.41)	0.030	0.505
Zambia RCT	1.48 (1.29, 1.70)	<0.001	0.443	1.34 (1.14, 1.57)	<0.001	0.484
DR Congo	1.24 (1.07, 1.43)	0.004	0.398	1.34 (1.09, 1.64)	0.005	0.398
India	1.04 (0.99, 1.10)	0.095	0.236	1.12 (1.03, 1.22)	0.009	0.192
Combined[c]	1.12 (1.07, 1.17)	<0.001	0.100	1.18 (1.10, 1.25)	<0.001	0.051
Combined Except India[c]	1.36 (1.25, 1.47)	<0.001	0.776	1.31 (1.18, 1.45)	<0.001	0.480

RCT= randomized, controlled trial

[a] All studies were adjusted for categorical ages (<5, 5-15, >15), treatment status (intervention/control) and, season (rainy/dry) except for Bolivia which was only adjusted for age because all observations occurred in the dry season. The Zambia Cross-Sectional Study is excluded from this table because there were no study arms and thus the results are the same as those reported in Table 5.

[b] P-values for the categorical study (control/intervention) parameter in each model

[c] Combined data includes the Zambia Cross-Sectional Study data as part of the control group
